# Cost-effectiveness analysis of bilateral cochlear implants for children with severe-to-profound sensorineural hearing loss in both ears in Singapore

**DOI:** 10.1371/journal.pone.0220439

**Published:** 2019-08-15

**Authors:** Li-Jen Cheng, Swee Sung Soon, David Bin-Chia Wu, Hong Ju, Kwong Ng

**Affiliations:** Agency for Care Effectiveness, Ministry of Health, Singapore, Singapore; University of Texas at Dallas, UNITED STATES

## Abstract

A cochlear implant is a small electronic device that provides a sense of sound for the user, which can be used unilaterally or bilaterally. Although there is advocacy for the benefits of binaural hearing, the high cost of cochlear implant raises the question of whether its additional benefits over the use of an acoustic hearing aid in the contralateral ear outweigh its costs. This cost-effectiveness analysis aimed to separately assess the cost-effectiveness of simultaneous and sequential bilateral cochlear implantations compared to bimodal hearing (use of unilateral cochlear implant combined with an acoustic hearing aid in the contralateral ear) in children with severe-to-profound sensorineural hearing loss in both ears from the Singapore healthcare payer perspective. Incremental quality-adjusted life year (QALYs) gained and costs associated with bilateral cochlear implants over the lifetime horizon were estimated based on a four-state Markov model. The analysis results showed that, at the 2017 mean cost, compared to bimodal hearing, patients receiving bilateral cochlear implants experienced more QALYs but incurred higher costs, resulting in an incremental cost-effectiveness ratio (ICER) of USD$60,607 per QALY gained for simultaneous bilateral cochlear implantation, and USD$81,782 per QALY gained for sequential bilateral cochlear implantation. The cost-effectiveness of bilateral cochlear implants is most sensitive to utility gain associated with second cochlear implant, and cost of bilateral cochlear implants. ICERs increased when the utility gain from bilateral cochlear implants decreased; ICERs exceeded USD$120,000 per QALY gained when the utility gain was halved from 0.03 to 0.015 in both simultaneous and sequential bilateral cochlear implantations. The choice of incremental utility gain associated with the second cochlear implant is an area of considerable uncertainty.

## Introduction

In Singapore, about 1.7 per 1,000 babies are diagnosed with severe-to-profound hearing loss or deafness at a median age of diagnosis of 2.7 months [1]. Hearing loss or deafness impacts language acquisition, overall literacy, development of social skills and attitudes including self-esteem in children [2]. The aim of overcoming hearing loss or deafness is to support functional hearing and meaningful speech perception and maximise the potential of living a productive life and successful integration into society. A cochlear implant is a small electronic device that stimulates the auditory nerve fibres directly to convey information to the brain on the amplitude and frequency of the sound signals [3], providing a sense of sound for the user who suffers from severe-to-profound sensorineural hearing loss [4].

The cochlear implant can be used alone as unilateral cochlear implant, combined with an acoustic hearing aid in the contralateral ear to give the bimodal configuration for hearing (bimodal hearing), or bilaterally. The use of cochlear implant can benefit incidental learning [5]. However, for children with poor residual hearing in the non-implanted ear, functional benefits may be limited. Research has shown that the inability to hear in only one ear can be detrimental to the child’s academic progression, self-esteem, and stress levels [6]. The failure to keep up academically may prompt a transfer from a mainstream school to a special school, which costs four times that of mainstream education in Singapore[7]. Although unilateral cochlear implant can provide substantial communication benefits compared to no cochlear implant,[8] children without additional disabilities receiving unilateral cochlear implant continue to fare worse than their normal hearing peers in academic performance, measured in written and oral language[9–11], mathematics[11], and grade failure rates[12].

In recent times, there has been emphasis on the benefits of binaural hearing, and many have advocated the use of a second cochlear implant in clinically appropriate candidates [13–15]. Compared to unilateral cochlear implant, bilateral cochlear implants have demonstrated advantages in sound localisation[16, 17], speech perception in noise[16, 17], complex language skills[17], hearing function in real-life situations[17], and mainstream school attendance[17]. Bilateral cochlear implants attempt to mimic binaural hearing (i.e. the use of both ears) like in normal hearing individuals. The ability to process sound signals from both ears can bring benefits through binaural squelch, head shadow effect and binaural redundancy[18]. The contralateral hearing aid used in bimodal hearing can give access to low-frequency speech signals which provide prosody and better speech recognition with competing talkers [19]. The combination of acoustic and electric stimulation in bimodal hearing has also been shown to give good music perception, and enjoyment of instrumental music [19]. Although bimodal hearing may give binaural hearing, for children with poor residual hearing in the non-implanted ear, functional benefits may be limited. If the hair cells are severely damaged, even large vibrations will not be converted to neural signals[20]. Unilaterally driven stimulation can lead to potentially irreversible reorganisation of the auditory cortex in the pathway deprived of stimulation. This can undermine integration and processing of auditory input, leading to asymmetric speech perception, poorer hearing in noise, abnormal sound localisation, and an inability to identify inter-aural timing cues. The amount of residual hearing needed in the un-implanted ear of bimodal hearing to restore binaural hearing remains unclear [21].

Bilateral cochlear implants potentially herald greater promise in binaural hearing. Bilateral cochlear implants can offer benefits over bimodal hearing in sound localisation and spatial unmasking [16, 17]. It is better than bimodal hearing in the ability to discriminate signal from noise through spatial unmasking and head shadow effect [22, 23] [24] [25], and in the gain in hearing ability from the use of second device (i.e. binaural advantage). Although some studies showed favourable speech perception in bilateral cochlear implants compared to bimodal hearing [26] [27] [25], others showed no difference [28]. There is inconsistent evidence of bilateral cochlear implants having advantages over bimodal hearing in vocabulary, sentence development, and language comprehension [29]. In addition, a meta-analysis comparing bilateral cochlear implants with bimodal hearing in speech recognition with noise found no difference in binaural summation and the head-shadow effect, with bilateral cochlear implants giving binaural squelch advantage [30]. The inconsistency in the incremental benefits of bilateral cochlear implants over bimodal hearing in auditory processing, functional hearing, and language development outcomes is a reflection that while restoring binaural hearing is the goal, this has not been completely realised yet [21].

As a high cost technology [16], the additional cost of the second cochlear implant raises the question of whether its additional benefits outweigh its costs. Economic evaluations, as comparative analyses of alternatives in terms of costs and consequences, have been used to inform healthcare resource allocation decisions. Cost-effectiveness analyses measure consequences in preference-based measures of health, with quality-adjusted life-year (QALY) as a common measure of effectiveness [31].

The aim of this study is to evaluate bilateral cochlear implants’ cost-effectiveness compared to bimodal hearing in children with severe-to-profound sensorineural hearing loss in both ears, from the Singapore healthcare payer perspective. As bilateral cochlear implants can take place simultaneously in a single surgical procedure (simultaneous bilateral cochlear implantation), or sequentially in two different surgical procedures (sequential bilateral cochlear implantation), both types of bilateral cochlear implantations are included as interventions and evaluated separately against bimodal hearing as the comparator in this study.

## Methods

The incremental cost-effectiveness ratio (ICER) is calculated by taking the incremental cost of intervention compared to comparator, divided by the incremental effectiveness of intervention compared to comparator [31]. This paper measures effectiveness in terms of QALY, which is derived by adjusting the length of a life-year with health utility scores corresponding to a health status. Health utility scores are measured on a scale of 0 corresponding to death, and 1 to perfect health [31].

### Patient population and settings

The eligible population for bilateral cochlear implants is children (<18 years old) with severe-to-profound sensorineural hearing loss in both ears. Children born without a cochlea or without auditory nerves, and those with neurological damage that prevent the processing of auditory information are not eligible for cochlear implants. This analysis uses bimodal hearing as the control group compared with: (1) simultaneous bilateral cochlear implantation: two initial cochlear implants received (one in each ear) at 1 year old; and separately (2) sequential bilateral cochlear implantation: one initial cochlear implant received at 1 year old and the second one in the contralateral ear two years later.

### Model structure

**Fig 1:**
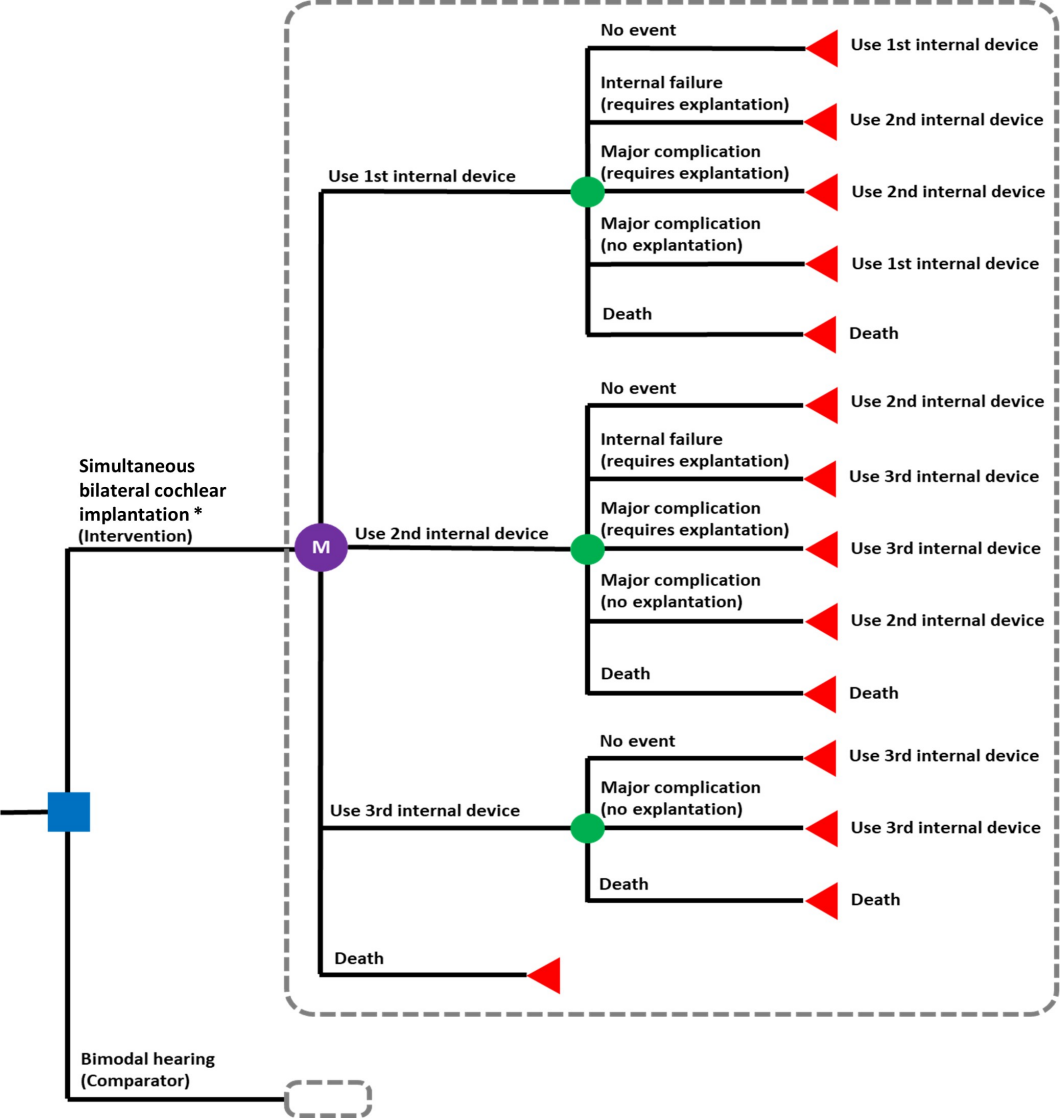
Cochlear implant model. * A separate pairwise comparison with bimodal hearing (comparator) was made using sequential bilateral cochlear implantation as the intervention.

A microsimulation model was developed to evaluate the cost-effectiveness of both simultaneous and sequential bilateral cochlear implantations compared to bimodal hearing as the control group in children born with severe-to-profound sensorineural hearing loss in both ears. A four-state Markov model was developed using TreeAge Pro 2016 (TreeAge Software, Inc., Massachusetts) in consultation with local experts to ensure face validity (see Fig 1). The four health states were “use of 1st internal device”, “use of 2nd internal device” (i.e. replacement device for the first internal device in the same ear), “use of 3rd internal device” (i.e. replacement device for the second internal device in the same ear), and “death” (i.e. all-cause mortality).

The model’s starting population comprises children who are eligible for bimodal hearing or bilateral cochlear implants. Eligible children enter the model at the health state “Use of 1st internal device” after the cochlear implantation. During the use of cochlear implant, children could develop clinical and device-related events, including internal device failure, major complications, wound revision due to major post-surgical complications, explantation and re-implantation, and all-cause mortality. They would either remain in the same health state, or transit to the health state “Use of 2nd internal device” if they experience an internal device failure or a major complication requiring explantation (followed by re-implantation). Similarly, should a second internal device failure or a major complication occur, they would transit to the health state “Use of 3rd internal device”.

Our model used a lifetime horizon and annual cycle length (includes half-cycle correction applied to QALY gained only). An annual discounting rate of 3% was applied to both costs and benefits. All analyses were conducted from the healthcare payer perspective. Monte Carlo simulation was used to run the analyses.

### Transition probabilities and assumptions

Hearing devices can experience device failure. As a cochlear implant consists of internal and external devices, device failures can be further separated into internal device failure or external device failure. Similar to hearing aid, replacement of the external cochlear implant device requires only a consultation with the audiologist without the need for surgery. Replacement of the internal cochlear implant device requires surgical explantation of the failed internal cochlear implant device and re-implantation of a new one. Transitions between health states in the model are due to internal device replacement as a result of major complications or internal device failure, or all-cause mortality [32, 33]. The list of transition probabilities, assumptions, and sources is presented in Table 1.

**Table 1:** Key model characteristics and assumptions.

Event	Variable		Value	Notes / Assumptions / Sources
**Major complications**	Probability of major complications	First year	year	A major complication is defined as an event requiring re-operation and hospitalisation: ◾ Surgical wound revision without explantation; ◾ Explantation and re-implantation of replacement internal device[16]. No surgical complication occurs after the initial implantation of the 1st cochlear implant in sequential bilateral cochlear implantation [34]. ^1^
		Subsequent years	0.001 / year	
	Probability of wound revision due to major complications	First year	0.005 / year	Of those experiencing major complications, it was assumed that half required surgical wound revision while the other half underwent explantation and re-implantation [16, 32]. Those requiring explantation will also undergo re-implantation.[16, 32]
		Subsequent years	0.0005 / year	
	Probability of explantation due to major complications	First year	0.005 / year	
		Subsequent years	0.0005 / year	
**Device failure**	Probability of internal cochlear implant device failure		Time-dependent	Failure rate will be reset upon internal device replacement due to either internal failure or major complications. Derived function for the probability of internal failure from the 20th to 80th year: 0.002911 x Nth Year of cochlear implant use + 0.998749 [35–37] A maximum of two cochlear implant internal device replacements during the simulated time horizon.
**Device upgrading**	External cochlear implant device		Every 7 years	Based on expert opinion, external processors need to be upgraded every 6 to 8 years because the technology becomes obsolete. Device is assumed to be upgraded every 7 years. ^1^
	Hearing aid		Every 5 years	Although the typical warranty periods for hearing aids are shorter than 5 years, based on expert opinion, a period of 5 years mirrors the average usage period before patient upgrades the device. ^1^
**All-cause mortality**	Background mortality		Age-dependent	Department of Statistics, Singapore[33]
**Others**	Age of 2nd cochlear implantation in sequential bilateral cochlear implantation		3 years old	The mean of between-implantation interval from 1 year to 5 years of age. The upper limit of 5 years old is determined by using 3.5 years old as critical age of development of cortical auditory activity, and between-implantation interval not exceeding 1.5 years [21] [38].
	Success of cochlear implantation surgeries		-	Based on expert opinion, failure of cochlear implantation surgery is very rare. It is reasonable to assume that all cochlear implantation surgeries are successful. ^1^
	Use of cochlear implant		-	Based on expert opinion, permanent voluntary non-use of a functioning cochlear implant is very unlikely to occur in Singapore. ^1^ Hence, the model did not consider voluntary non-use of a functioning cochlear implant as a modelling parameter.

Where assumptions were based on expert opinion, expert opinion was sought from clinicians, audiologists, auditory-verbal therapists, medical social workers, and educators.

The reliability of cochlear implant refers to the length of time for which implants work before they need replacement. We used empirical survival data from the manufacturers’ cochlear implant reliability reports to derive the probability of cochlear implant internal device failure [35–37]. All internal devices currently offered commercially by the three major manufacturers are considered to have similar aggregated lifetimes regardless of brands and models. The follow-up data are available for 19 years post-initial cochlear implantation. A linear regression function was fitted to extrapolate the probability of cochlear implant internal failure from the 19th year onwards.

### Utility/Health outcomes

The health outcome measure is the incremental QALYs gained associated with bilateral cochlear implants use over a lifetime horizon. In the absence of local data, we applied the same method, values, and assumptions as Bond et al. (2009)[16], with the incremental health utility of +0.232 for bimodal (versus no implantation) [39], and +0.03 for bilateral cochlear implants (versus bimodal) [40]. These increments, taken from adults, are associated with improvements in quality of life underpinned by improvements in sound localisation and speech perception in noise [40]. We assumed that full utility gains can be realised only from the ages between 4 and 54 years old [16]. From ages 1 to 3 years old, the utility gain is lower as the full benefits of cochlear implant take time to develop; from 55 years old onwards, a declining utility gain was applied to reflect aging in general population [39]. Table 2 shows the changing annual incremental utility gains across different age bands.

**Table 2:** Utility values by age.

	Age band (in years)							
	1	2–3	4–54	55–64	65–69	70–74	75–79	80–84
**Scaling factor**	28%	91%	100%	98%	96%	91%	84%	72%
**Utility gain (Bimodal)**	0.066	0.212	0.232	0.227	0.223	0.211	0.195	0.167
**Utility gain (Bilateral cochlear implants versus Bimodal)**	0.009	0.027	0.03	0.0294	0.0288	0.0273	0.0252	0.0216

### Resource use and cost

As the clinical pathways for children receiving bilateral cochlear implants and bimodal hearing are the same for pre-surgery assessment for the second cochlear implant in both simultaneous and sequential bilateral cochlear implants, and post-surgery switch-on, mapping, and habilitation, only incremental resource use and costs relative to bimodal hearing were included in the analyses. This is similar to the approach by Martin et al. (2017) which compared bilateral cochlear implantation with unilateral cochlear implantation [41]. Local cochlear implant experts indicated that compared to children receiving a single cochlear implant in bimodal hearing, children receiving simultaneous bilateral cochlear implantation will undergo a longer surgery procedure, with the same the intensity of follow-up reviews, mapping, and habilitation sessions. In sequential bilateral cochlear implantation, resource use is doubled since the child has to undergo additional pre-surgery assessment, surgery, and post-surgery habilitation sessions. During the between-implantation interval in sequential bilateral cochlear implantation, the child will continue to use a hearing aid in the contralateral ear. For cochlear re-implantation, the explantation and re-implantation process generally take place in the same surgical session. We assumed that the two implants in bilateral cochlear implants are independent of each other. As the probabilities of any major complication or device failure are the same in both ears, costs relating to replacements for bilateral cochlear implants (e.g. cochlear implant devices, cochlear implantation procedure, complication management) were assumed to be twice that of patients with bimodal hearing.

All relevant unit charges were obtained from manufacturer and public healthcare institutions in 2017. They were assumed to remain constant over the simulated model period.

The units of resource use were determined with input from local experts, and they are shown in Table 3. All cost-related model parameters are listed in Table 4. For cochlear implant device cost variables, we used the 2017 mean cost of cochlear implant in Singapore. All costs are expressed in 2017 US dollars ($, USD1 = SGD1.317).

**Table 3:** Units of incremental resource use comparing bilateral cochlear implantation intervention groups to bimodal hearing control group.

Description of resource	Year of resource use in model	Comparison	Differences in resource use													Additional remarks
			Audiologist review	Auditory assessment / aided hearing test	Auditory-verbal therapist / speech language pathologist review	Ear, nose, throat surgeon review	Speech assessment	Computerised tomography scan	Cochlear implant device	Surgery for cochlear implantation in one ear	Hospitalisation in general ward	Surgery for wound revision / explantation and re-implantation in one ear	Internal cochlear implant device replacement	External cochlear implant device replacement	Hearing aid	
**Pre-surgery assessment for the second cochlear implant**	2nd year (i.e. 1 year after implantation of first cochlear implant in sequential bilateral cochlear implantation)	Simultaneous bilateral cochlear implantation vs bimodal hearing	No difference since pre-surgery assessment for second cochlear implant is done together with the first cochlear implant.						Not applicable in pre-surgery assessment.							No additional remarks
		Sequential bilateral cochlear implantation vs bimodal hearing	Sequential bilateral cochlear implantation requires more resources than bimodal hearing:						Not applicable in pre-surgery assessment.							
			4 sessions more	4 sessions more	6 sessions more	2 sessions more	1 session more	1 scan more								
**Initial implantation of second cochlear implant**	1st year	Simultaneous bilateral cochlear implantation vs bimodal hearing	Not applicable in initial implantation of second cochlear implant.						Simultaneous bilateral cochlear implantation requires more resources than bimodal hearing:		Not applicable in initial implantation of second cochlear implant.					No additional remarks
									1 device more	1 surgery more						
		Sequential bilateral cochlear implantation vs bimodal hearing	Not applicable in initial implantation of second cochlear implant.						Not applicable as second cochlear implant is implanted only in 3rd year in model.		Not applicable in initial implantation of second cochlear implant.					
	3rd year	Simultaneous bilateral cochlear implantation vs bimodal hearing	Not applicable in initial implantation of second cochlear implant.						Not applicable as second cochlear implant is implanted in 1st year in model.		Not applicable in initial implantation of second cochlear implant.					
		Sequential bilateral cochlear implantation vs bimodal hearing	Not applicable in initial implantation of second cochlear implant.						Sequential bilateral cochlear implantation requires more resources than bimodal hearing:			Not applicable in initial implantation of second cochlear implant.				
									1 device more	1 surgery more	1 day more					
**Post-surgery switch-on, mapping & habilitation**	3rd year (i.e. same year as implantation of second cochlear implant in sequential bilateral cochlear implantation)	Simultaneous bilateral cochlear implantation vs bimodal hearing	No difference as simultaneous bilateral cochlear implantation has the same resource utilisation as bimodal hearing.					Not applicable in post-surgery switch-on, mapping & habilitation.								Based on expert opinion, additional resources for habilitation post initial implantation of second cochlear implant in sequential bilateral cochlear implantation apply for 3 years (including year of implantation of second cochlear implant). Mapping sessions and auditory-verbal therapist / speech language pathologist reviews were most intensive in the period immediately after implantation and taper thereafter.
		Sequential bilateral cochlear implantation vs bimodal hearing	Sequential bilateral cochlear implantation requires more resources than bimodal hearing:					Not applicable in post-surgery switch-on, mapping & habilitation.								
			10 sessions more (includes mapping)	2 sessions more	52 sessions more	2 sessions more	2 sessions more									
	4th year (i.e. 1 year after implantation of second cochlear implant in sequential bilateral cochlear implantation)	Simultaneous bilateral cochlear implantation vs bimodal hearing	No difference as simultaneous bilateral cochlear implantation has the same resource utilisation as bimodal hearing.					Not applicable in post-surgery switch-on, mapping & habilitation.								
		Sequential bilateral cochlear implantation vs bimodal hearing	Sequential bilateral cochlear implantation requires more resources than bimodal hearing:					Not applicable in post-surgery switch-on, mapping & habilitation.								
			2 sessions more (includes mapping)	2 sessions more	26 sessions more	1 session more	2 sessions more									
	5th year (i.e. 2 years after implantation of second cochlear implant in sequential bilateral cochlear implantation)	Simultaneous bilateral cochlear implantation vs bimodal hearing	No difference as simultaneous bilateral cochlear implantation has the same resource utilisation as bimodal hearing.					Not applicable in post-surgery switch-on, mapping & habilitation.								
		Sequential bilateral cochlear implantation vs bimodal hearing	Sequential bilateral cochlear implantation requires more resources than bimodal hearing:					Not applicable in post-surgery switch-on, mapping & habilitation.								
			2 sessions more (includes mapping)	2 sessions more	2 sessions more	1 session more	2 sessions more									
**Major complications involving wound revision without explantation**	When wound-related complication occurs	Simultaneous bilateral cochlear implantation vs bimodal hearing	Not applicable to major complications involving wound revision without explanation.								Simultaneous bilateral cochlear implantation requires more resources than bimodal hearing:		Not applicable to major complications involving wound revision without explanation.			No additional remarks
											1 day more	1 surgery more (wound revision)				
		Sequential bilateral cochlear implantation vs bimodal hearing	Not applicable to major complications involving wound revision without explanation.								Sequential bilateral cochlear implantation requires more resources than bimodal hearing:		Not applicable to major complications involving wound revision without explanation.			
											1 day more	1 surgery more (wound revision)				
**Major complications involving explantation and re-implantation**	When major complication involving explantation occurs	Simultaneous bilateral cochlear implantation vs bimodal hearing	Not applicable to major complications involving explantation and re-implantation.								Simultaneous bilateral cochlear implantation requires more resources than bimodal hearing:			Not applicable to major complications involving explantation and re-implantation.		Internal cochlear implant device replacement cost applies only after 10-year warranty period.
											1 day more	1 surgery more (explantation and re-implantation)	1 device more			
		Sequential bilateral cochlear implantation vs bimodal hearing	Not applicable to major complications involving explantation and re-implantation.								Sequential bilateral cochlear implantation requires more resources than bimodal hearing:			Not applicable to major complications involving explantation and re-implantation.		
											1 day more	1 surgery more (explantation and re-implantation)	1 device more			
**Routine external cochlear implant device replacement**	Every 7 years	Simultaneous bilateral cochlear implantation vs bimodal hearing	Not applicable to routine external cochlear implant device replacement.											Simultaneous bilateral cochlear implantation requires more resources than bimodal hearing:	Not applicable to routine external cochlear implant device replacement.	No additional remarks
														1 device more		
		Sequential bilateral cochlear implantation vs bimodal hearing	Not applicable to routine external cochlear implant device replacement.											Sequential bilateral cochlear implantation requires more resources than bimodal hearing:	Not applicable to routine external cochlear implant device replacement.	
														1 device more		
**Routine hearing aid replacement**	Every 5 years	Simultaneous bilateral cochlear implantation vs bimodal hearing	Not applicable to routine hearing aid replacement.												Simultaneous bilateral cochlear implantation requires more resources than bimodal hearing:	Hearing aid replacement incurs only in bimodal hearing for each pairwise comparison.
															1 device more	
		Sequential bilateral cochlear implantation vs bimodal hearing	Not applicable to routine hearing aid replacement.												Sequential bilateral cochlear implantation requires more resources than bimodal hearing:	
															1 device more	

Wound revision surgery includes flap closure surgery, repair of cerebrospinal fluid leakage surgery, and wound debridement.

**Table 4:** Cost parameters of resource use.

Resource type	Unit	Per unit cost ($, USD)
**Surgery**		
**- Cochlear implantation in one ear**	Surgery	4,940
**- Wound revision without explantation in one ear**	Surgery	6,454
**- Explantation and re-implantation in one ear**	Surgery	7,115
**Hospitalisation**		
**- General ward**	Day	172
**Device**		
**- Cochlear implant device**	Device	28,216
**- Hearing aid**	Device	949
**Assessment & habilitation**		
**- Aided hearing test**	Assessment	29
**- Audiologist review (includes mapping)**	Session	81
**- Auditory assessment**	Assessment	53
**- Auditory-verbal therapist / speech language pathologist review**	Session	116
**- Computerised tomography scan**	Scan	405
**- Ear, nose, throat surgeon review**	Session	57
**- Speech assessment**	Assessment	35

Wound revision surgery includes flap closure surgery, repair of cerebrospinal fluid leakage surgery, and wound debridement.

### Deterministic one-way sensitivity analysis

Deterministic one-way sensitivity analyses were conducted over the range of predefined values of the point estimates for model parameters (i.e. ±10% or feasible range) separately for simultaneous and sequential bilateral cochlear implantation. Since all simulated cochlear implant users would receive a replacement internal cochlear implant device following internal device failure or a major complication requiring explantation (and re-implantation), these variables were not assumed to alter the cochlear implant users’ utility except after transiting to the death state. The impact of replacing an acoustic hearing aid or the external cochlear implant device on utility levels was taken to be minimal due to the transient nature of replacement. The parameters included in the one-way sensitivity analyses were utility gain from the second cochlear implant in both simultaneous and sequential bilateral cochlear implantations, acquisition cost of cochlear implant system, cost of hearing aid, cost of cochlear implantation in one ear and cost of re-implantation. Results were plotted as a Tornado diagram with the variables arranged in descending order according to the extent of the parameter’s impact on the ICER.

### Probabilistic sensitivity analysis

A multivariate probabilistic sensitivity analysis was performed, using 10,000 second-order Monte Carlo simulation. We assigned gamma distributions to all cost variables and beta distribution to utility variables, and assumed that the standard deviation of each parameter was set to its mean. As Singapore does not have an explicit willingness-to-pay threshold to determine whether a health technology represents good value for money, cost-effectiveness acceptability curves were generated to present the probability of two types of bilateral cochlear implantations and bimodal hearing being cost-effective at varying willingness-to-pay thresholds.

## Results

### Base case analysis

In the base case analysis, compared to the control group (bimodal hearing), the ICERs for simultaneous and sequential bilateral cochlear implantations are $60,607 and $81,782 per QALY gained respectively (Table 5).

**Table 5:** Base case analysis results.

	Control		Intervention			
	Bimodal hearing		Simultaneous bilateral cochlear implantation		Sequential bilateral cochlear implantation	
	Cost ($)	QALY	Cost ($)	QALY	Cost ($)	QALY
**Mean Values**	81,260	6.82	134,710	7.70	148,793	7.64
**Incremental Values**	-		53,451	0.88	67,533	0.83
**ICER (cost/QALY)**	-		60,607		81,782	

ICER, incremental cost-effectiveness ratio; QALY, quality-adjusted life year

### One-way sensitivity analysis

**Fig 2:**
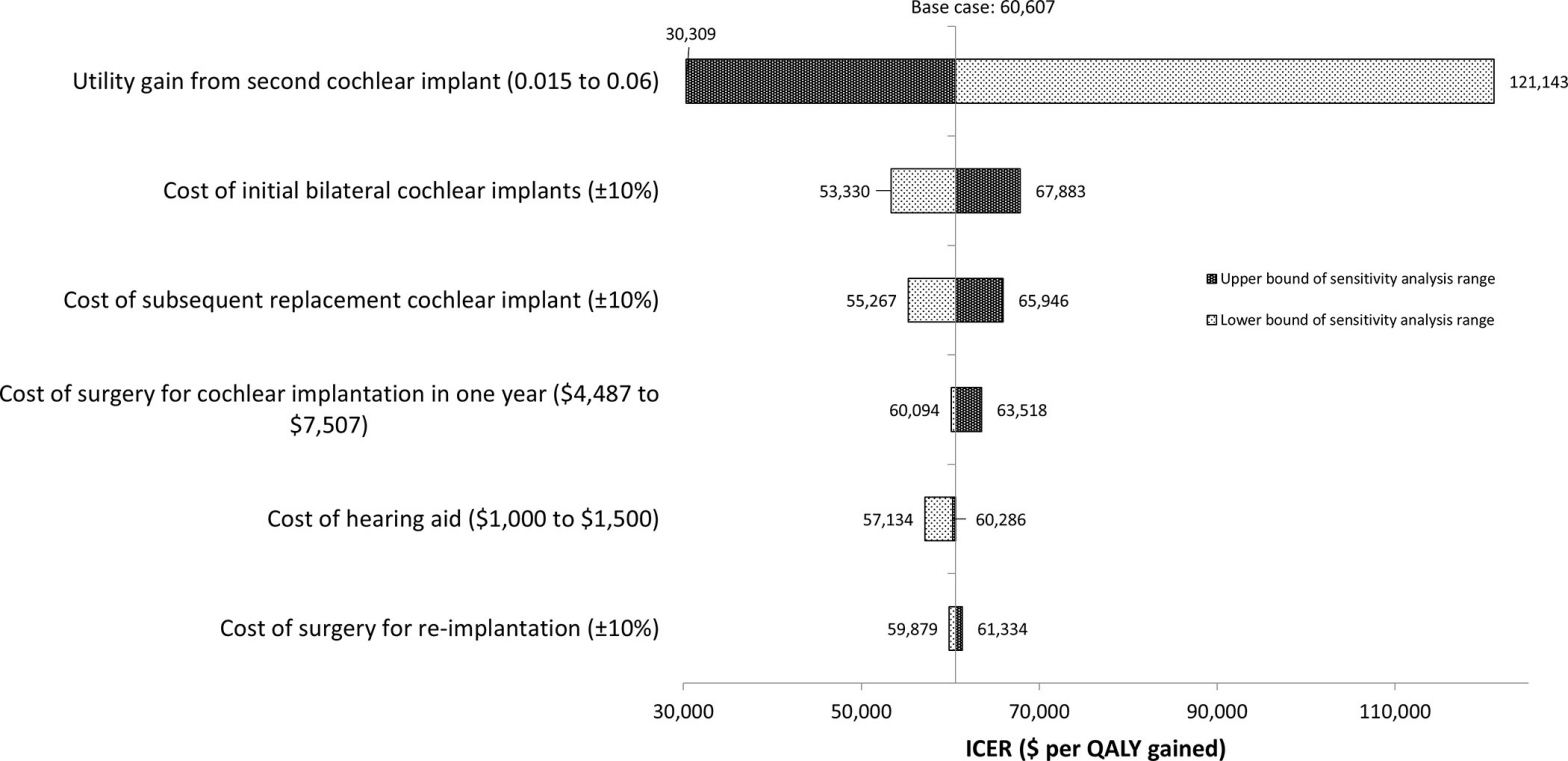
One-way sensitivity analysis results (simultaneous bilateral cochlear implantation). ICER, incremental cost-effectiveness ratio; QALY, quality-adjusted life year.

We performed one-way sensitivity analyses to identify the variables that impacted the ICERs the most. Our results showed that when simultaneous and sequential bilateral cochlear implantations were independently compared to bimodal hearing, the ICERs were most sensitive to the utility gain from the second cochlear implant and the cost of initial bilateral cochlear implants. When the incremental utility gain for the second cochlear implant was halved from 0.03 to 0.015, the ICER of simultaneous bilateral cochlear implantation almost doubled to $121,143 per QALY gained. When the cost of the initial bilateral cochlear implantation increased by 10%, the ICER of simultaneous bilateral cochlear implantation was $67,883 per QALY gained; when the cost was reduced by 10%, the ICER of simultaneous bilateral cochlear implantation was $53,330 per QALY gained (see Fig 2). Other cost variables on surgery for cochlear implantation, hearing aid, and surgery for re-implantation had a relatively much smaller impact on the ICERs ranging from -6% to +5% change in the base case ICER when simultaneous bilateral cochlear implantation was compared to bimodal hearing.

**Fig 3:**
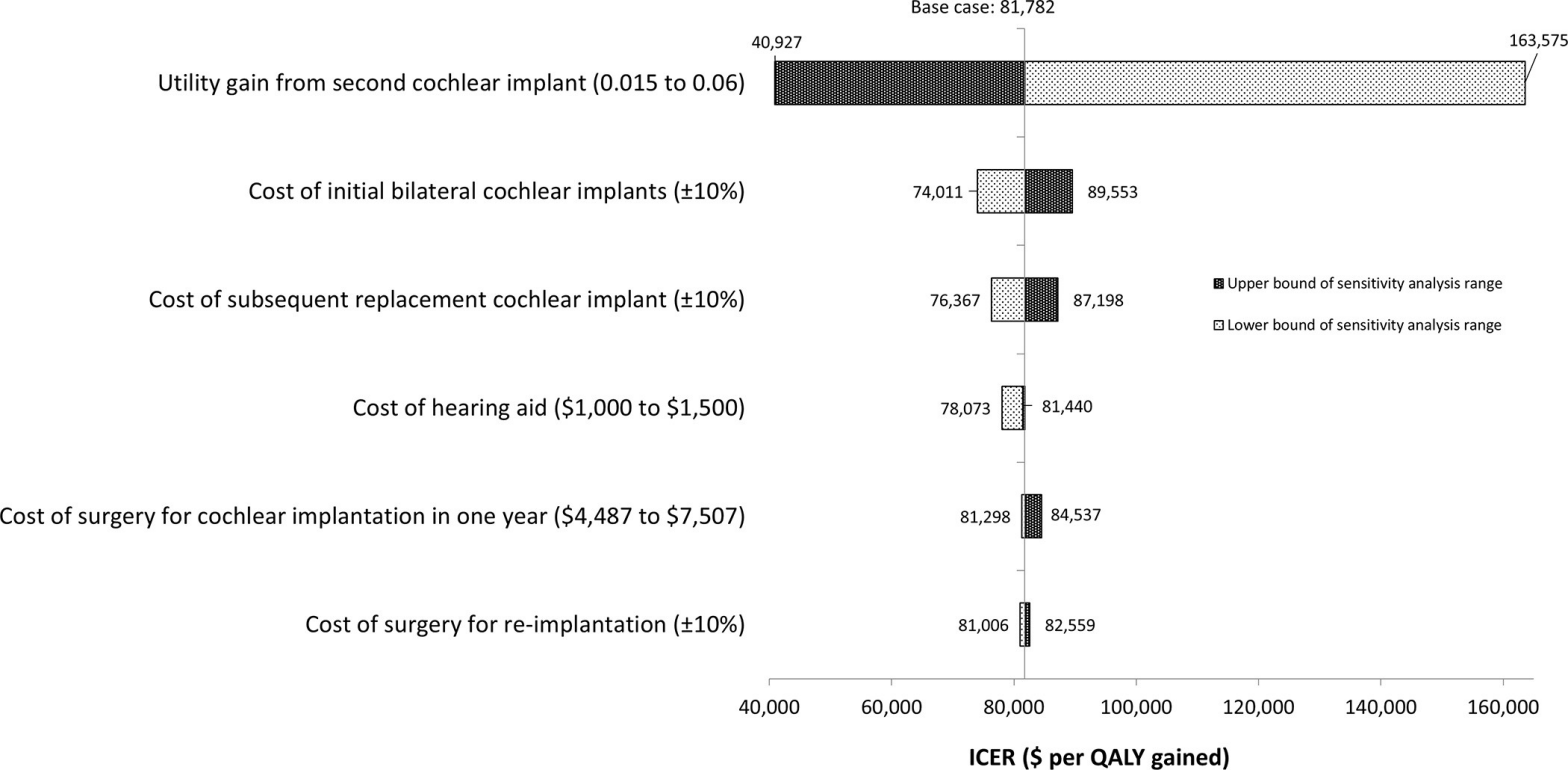
One-way sensitivity analysis results (sequential bilateral cochlear implantation). ICER, incremental cost-effectiveness ratio; QALY, quality-adjusted life year.

For sequential bilateral cochlear implantation, the ICER increased to $163,575 per QALY gained when the incremental utility gain for the second cochlear implant was halved from 0.03 to 0.015, and to $89,553 per QALY gained when the cost of initial bilateral cochlear implants increased by 10%. When the cost was reduced by 10%, the ICER of sequential bilateral cochlear implantation reduced to $74,011 per QALY gained (see Fig 3). Similar to simultaneous bilateral cochlear implantation, variables not relating to utility gain from second cochlear implant and cost of cochlear implants had much smaller impact on the ICERs, ranging from -5% to +3% change in base case ICER when sequential bilateral cochlear implantation was compared to bimodal hearing.

### Probabilistic sensitivity analysis

**Fig 4:**
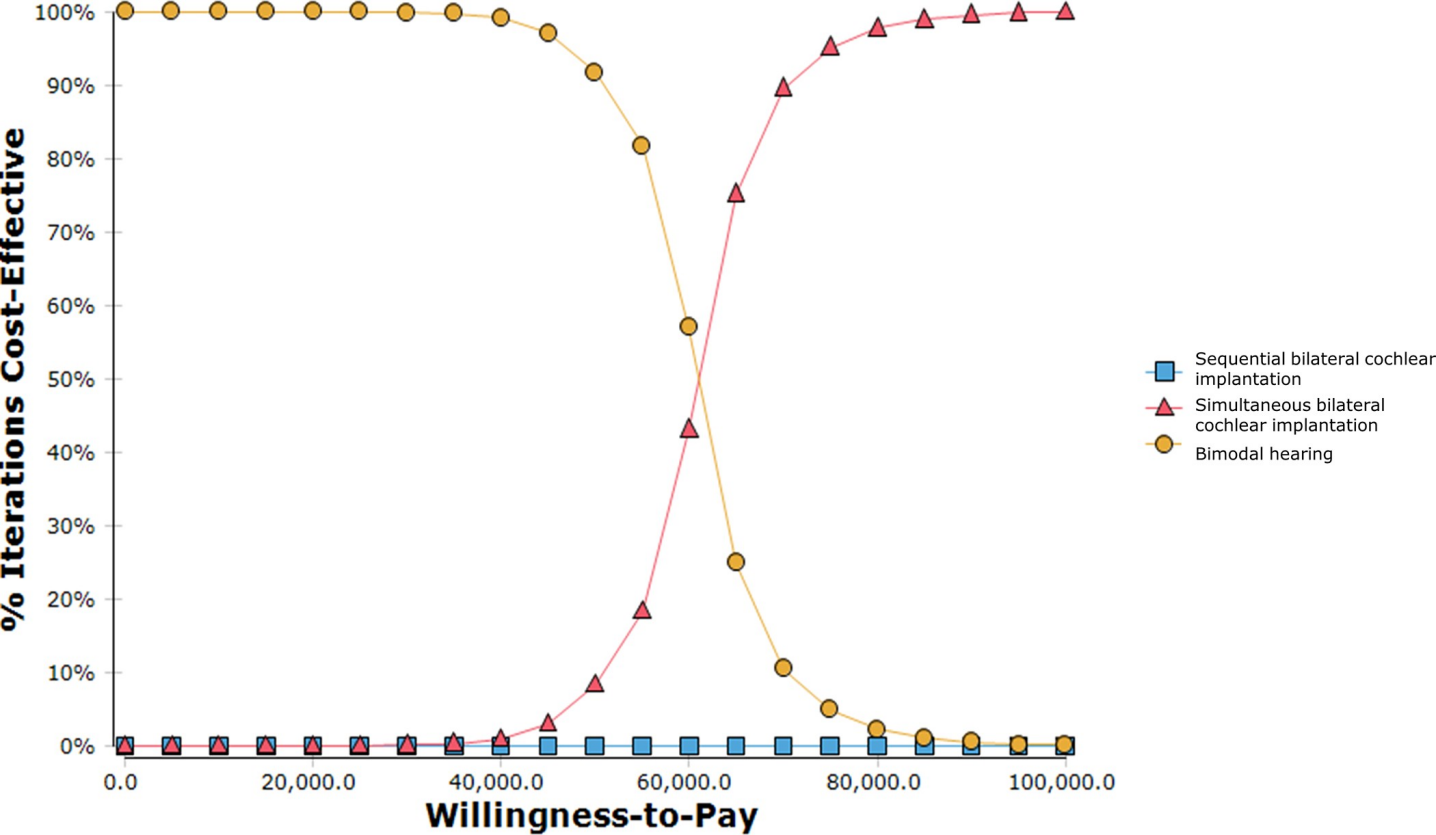
Cost-effectiveness acceptability curve.

The average costs and QALYs over the 10,000 Monte Carlo simulations were essentially similar to the deterministic base case results. The mean probabilistic ICER was $60,995 and $82,111 per QALY gained for simultaneous and sequential bilateral cochlear implantations respectively when they were separately compared to bimodal hearing. The Monte Carlo simulation results are presented as cost-effectiveness acceptability curves in Fig 4. It showed that the curves for simultaneous bilateral cochlear implantation and bimodal hearing crossed at a willingness-to-pay of about $61,000 per QALY gained. When willingness-to-pay was less than $61,000, bimodal hearing was the most cost-effective option; when willingness-to-pay exceeded $61,000, simultaneous bilateral cochlear implantation became increasingly cost-effective. Sequential bilateral cochlear implantation was dominated (i.e. more costly with lesser QALYs gained) by simultaneous bilateral cochlear implantation and bimodal hearing when willingness-to-pay was varied from $0 to $100,000 per QALY gained.

## Discussion

Results from our cost-effectiveness model were fairly consistent with published economic evaluations on bilateral cochlear implantation in populations comprising 80% bimodal hearing users—Bond et al. (2009) [16] and Summerfield et al. (2010) [42]. Both studies showed that in the UK context, bilateral cochlear implants could be a cost-effective option when annual utility increment was increased from +0.03 to +0.06 or when there was a price discount of at least 60% for the second cochlear implant, underscoring how sensitive the ICERs were to these two variables. The use of incremental utility gain of +0.03 in Bond et al (2009) for the second cochlear implant resulted in an ICER of £40,410 (QALY gain: 0.67) for simultaneous bilateral cochlear implantation, while that of +0.063 in Summerfield et al. (2010) gave an ICER of £21,768 (QALY gain: 1.57)[42]. When Bond et al. (2009) raised the annual utility increment to 0.06, a similar ICER (£21,526 per QALY gained) was reported[16].

The choice of incremental utility gain associated with the second cochlear implant, whether for simultaneous or sequential bilateral cochlear implantation, is an area of considerable uncertainty. Previous studies have demonstrated greater gains associated with unilateral cochlear implant than bilateral hearing aids in hearing, speech perception and speech production, enabling children with profound hearing loss or deafness to interpret sounds, understand other people, be better understood, and function more safely in their environment [43]. The impact of these benefits on daily life justifies the greater gain in health utility (+0.232), which was based on the Health Utilities Index Mark 3, obtained from a regression analysis of a sample of 403 implanted children [39]. In contrast, functional improvements that commensurate with incremental utility gains with the second cochlear implant in bilateral cochlear implantation are less clear. In the absence of randomised clinical trial evidence, as with Bond et al. (2009), the incremental utility gain of +0.03 applied in the base case was obtained from a small sample of 24 adult bilateral cochlear implants users in the UK [40]. The wide 95% confidence interval reported in the study ranged from -0.045 to +0.104, possibly due to the small sample. The use of utility gain estimates from a small sample of adults may not be readily generalisable to the paediatric population. In our sensitivity analyses, we had used a utility gain of +0.06 from Summerfield et al. (2010) where 180 informants comprising clinicians, researchers, students, and parents valued the quality of life of a hypothetical child born profoundly deaf [42]. Although the time trade-off data found that bilateral cochlear implants when compared with bimodal hearing was associated with an incremental utility of +0.063, the distributions of the increments were broad and skewed, with a third of the informants giving negative or zero. In view of the paucity of studies with reliable utility gain values and the lack of robust clinical evidence in showing the incremental benefits of bilateral cochlear implantation over bimodal hearing, a more conservative utility gain of +0.03 was used in the base case to avoid underestimating the ICERs.

A recently published systematic review concluded that there was insufficient evidence to demonstrate consistent benefits when comparing bimodal hearing with simultaneous or sequential bilateral cochlear implantation in children [44]. Although the evidence showed that bilateral cochlear implants did not differ from bimodal hearing in terms of language development and speech perception, evidence for sound localisation favoured the bilateral cochlear implantation group. The inconsistent clinical benefits made it difficult to understand how they translate into the overall well-being and development of the individual. Although the ultimate aim of restoring hearing loss or deafness is to maximise the potential of the individual, there remains a gap in the evidence on the incremental difference in academic performance, and career achievements or progression between bilateral cochlear implants and bimodal hearing. Given the lack of consistent evidence demonstrating that bilateral cochlear implants is functionally superior to bimodal hearing, a conservative utility gain estimate of +0.03 was used in our model. Local clinicians indicated that most children with bimodal hearing are able to attend mainstream schools, further strengthening the case for using a more modest utility gain estimate. Given the considerable uncertainty surrounding the available evidence on utility gain, lowering the cost of bilateral cochlear implants would be the appropriate measure to improve its cost-effectiveness compared with bimodal hearing. Bond et al. (2009) found that at a willingness-to-pay threshold of £30,000 per QALY, simultaneous bilateral cochlear implantation became cost-effective when a discount of approximately 60% is offered on the second cochlear implant in bilateral cochlear implants. In our model, a reduction of 10% in cost of initial bilateral cochlear implants led to a corresponding 10 to 12% reduction in the base case ICERs, indicating reducing the cochlear implant cost as a practical method to improve the cost-effectiveness of simultaneous and sequential bilateral cochlear implantations.

Results from the probabilistic sensitivity analysis yielded cost-effectiveness acceptability curves that showed that sequential bilateral cochlear implantation was dominated throughout the willingness-to-pay from $0 to $100,000 per QALY gained. This suggests that a delay of merely two years during the critical age of learning could diminish the accrual of benefits in QALYs to an extent that renders sequential bilateral cochlear implantation not cost-effective throughout the willingness-to-pay range. Sequential bilateral cochlear implantation was dominated by simultaneous bilateral cochlear implantation, mainly due to its slower accrual of QALYs and higher post-surgery habilitation costs.

There are several limitations in this study. The first limitation relates to uncertainties regarding the real incremental benefits of the second cochlear implant in both simultaneous and sequential bilateral cochlear implantations compared with bimodal hearing. In light of the considerable uncertainty surrounding the choice of incremental utility gain, and the lack of supporting evidence, we were unable to incorporate the effects of education and career achievement, whether in the form of changes in utility gain or productivity loss. More longitudinal research in paediatric patients comparing bimodal hearing with bilateral cochlear implants in utility changes and clinically-relevant long-term outcomes that track functional performance, psychosocial well-being, and achievements in academics and career will help to mitigate the uncertainty.

Second, we are aware that a constant utility gain over a long time horizon may conceal possible changes in utility associated with hearing loss or deafness as a result of severity of condition and psychosocial adjustment over time. This may potentially overestimate the benefits of the second cochlear implant in both simultaneous and sequential bilateral cochlear implantations for children.

Third, as the utility scores reported in existing literature were generated in western countries, it is unclear if they could be readily generalised to the Singapore context, particularly if they have communities with a stronger Deaf culture, or different perception of benefits across cultural groups, such as the child’s academic progression, self-esteem, and stress levels.

Fourth, device failure rates were derived from survival data of all internal devices found in manufacturers’ online reliability reports, with follow-up periods ranging from 2 to 19 years. The earlier the device was launched, the longer their survival data, which implied that a greater weight was conferred to older models in the estimation of reliability. Yet, with continuous advancements in cochlear implant technologies, the more recently launched cochlear implants are expected to have a lower failure rate. This could potentially overestimate the average failure rate of the internal device. However, since only two internal cochlear implant device replacements were allowed over the simulated lifetime in the model, the impact of this overestimation on the ICERs would be negligible.

Fifth, we were unable to account for the effects of any incremental innovation associated with newer hearing aid technologies. Hearing aids vary in the number of sound processing channels and how well they can filter background noises to enhance speech signals. When used in the bimodal way, advanced hearing aids are equipped with features to share sound signals received by both devices to enhance the binaural experience [45, 46]. The evolving innovation of hearing aid technologies may potentially narrow the gap between bimodal hearing and bilateral cochlear implants, and any resultant incremental utility gain.

## Conclusion

In children with severe-to-profound sensorineural hearing loss, compared to bimodal hearing, simultaneous bilateral cochlear implantation gave an ICER of $60,607 per QALY gained, while sequential bilateral cochlear implantation gave an ICER of $81,782 per QALY gained. Sensitivity analyses found that the results were most sensitive to utility gain associated with the second cochlear implant, and cost of bilateral cochlear implants. The choice of incremental utility gain associated with the second cochlear implant, whether for simultaneous or sequential bilateral cochlear implantation, is an area of considerable uncertainty. In view of the considerable uncertainty in the incremental clinical benefits, bilateral cochlear implantation may not represent good value for limited healthcare dollars in Singapore context unless the cost of bilateral cochlear implants is appropriately reduced.

## Supporting information

S1 File SupportingInfo_CI survival data. (DOCX)

## References

[1] Low W.K., et al, Universal newborn hearing screening in Singapore: the need, implementation and challenges. Ann Acad Med Singapore, 2005 34(4): p. 301–6. 15937570

[2] World Health Organization, *Childhood hearing loss*. 2016, World Health Organization (WHO): Geneva, Switzerland.

[3] Loizou, P., Mimicking the human ear IEEE Signal Processing Magazine, 1998: p. 101–130.

[4] National Institute on Deafness and Other Communication Disorders (NIDCD). NIDCD Fact Sheet—Cochlear Implants. 2016; Available from: https://www.nidcd.nih.gov/sites/default/files/Documents/health/hearing/FactsheetCochlearImplants.pdf.

[5] De Raeve L., Vermeulen A., and Snik A., Verbal cognition in deaf children using cochlear implants: effect of unilateral and bilateral stimulation. Audiol Neurootol, 2015 20(4): p. 261–6. 10.1159/000381003 26021884

[6] Kuppler K., Lewis M., and Evans A.K., *A review of unilateral hearing loss and academic performance: is it time to reassess traditional* dogmata? Int J Pediatr Otorhinolaryngol, 2013 77(5): p. 617–22. 10.1016/j.ijporl.2013.01.014 23474216

[7] Ministry of Health, *Report of the Committee to study the early detection and treatment of hearing loss in children in Singapore*. 2001, Ministry of Health: Singapore.

[8] Beadle E.A.R., et al, Long-term functional outcomes and academic-occupational status in implanted children after 10 to 14 years of cochlear implant use. Otol Neurotol, 2005 26: p. 1152–60. 16272934 10.1097/01.mao.0000180483.16619.8f

[9] Geers A.E., et al, Spoken language scores of children using cochlear implants compared to hearing age-mates at school entry. Journal of Deaf Studies and Deaf Education, 2009 14(3): p. 371–385. 10.1093/deafed/enn046 19155289

[10] Huber M. and Kipman U., Cognitive skills and academic achievement of deaf children with cochlear implants. Otolaryngology—Head and Neck Surgery (United States), 2012 147(4): p. 763–772. 10.1177/0194599812448352 22623402

[11] Sarant J.Z., Harris D.C., and Bennet L.A., Academic outcomes for school-aged children with severe–profound hearing loss and early unilateral and bilateral cochlear implants. Journal of Speech, Language, and Hearing Research, 2014 58: p. 1017–32. 10.1044/2015_JSLHR-H-14-0075 25677804

[12] Venail F., et al, Educational and employment achievements in prelingually deaf children who receive cochlear implants. Archives of Otolaryngology—Head and Neck Surgery, 2010 136(4): p. 366–372. 10.1001/archoto.2010.31 20403853

[13] Offeciers E., et al, International consensus on bilateral cochlear implants and bimodal stimulation. Acta Otolaryngol, 2005 125(9): p. 918–9. 10.1080/00016480510044412 16109670

[14] Ramsden J.D., et al, European Bilateral Pediatric Cochlear Implant Forum consensus statement. Otol Neurotol, 2012 33(4): p. 561–5. 10.1097/MAO.0b013e3182536ae2 22569146

[15] Balkany T., et al, William House Cochlear Implant Study Group: position statement on bilateral cochlear implantation. Otol Neurotol, 2008 29(2): p. 107–8. 10.1097/mao.0b013e318163d2ea 18223440 PMC2701670

[16] Bond M., et al, The effectiveness and cost-effectiveness of cochlear impalnts for severe to profound deafness in children and adults: a systematic review and economic model. Health Technology Assessment, 2009 13(44): p. 1–330. 10.3310/hta13440 19799825

[17] Washington State Health Care Authority, *Cochlear Implants: Bilateral versus Unilateral—Final Evidence Report*. 2013, Washington State Health Care Authority: PA.

[18] Dowell R., et al Bilateral Cochlear Implants in Children. Seminars in Hearing, 2011 32(1): p. 53–72.

[19] Cullington H.E. and Zeng F.G., Comparison of bimodal and bilateral cochlear implant users on speech recognition with competing talker, music perception, affective prosody discrimination, and talker identification. Ear Hear, 2011 32(1): p. 16–30. 10.1097/AUD.0b013e3181edfbd2 21178567 PMC3059251

[20] National Institute on Deafness and Other Communication Disorders (NIDCD). NIDCD Fact Sheet—Hearing Aids. 2015 [cited 2017 2 Aug]; Available from: https://www.nidcd.nih.gov/sites/default/files/Documents/health/hearing/nidcd-hearing-aids.pdf.

[21] Gordon K.A., Jiwani S., and Papsin B.C., Benefits and detriments of unilateral cochlear implant use on bilateral auditory development in children who are deaf. Frontiers in Psychology, 2013 4(719). 10.3389/fpsyg.2013.00719 PMC3797443 24137143

[22] Mok M., et al, Spatial unmasking and binaural advantage for children with normal hearing, a cochlear implant and a hearing aid, and bilateral implants. Audiology and Neurotology, 2007 12(5): p. 295–306. 10.1159/000103210 17536198

[23] Mok M., et al, Speech perception benefit for children with a cochlear implant and a hearing aid in opposite ears and children with bilateral cochlear mplants. Audiology and Neurotology, 2010 15(1): p. 44–56. 10.1159/000219487 19468210

[24] Litovsky R.Y., et al, Bilateral cochlear implants in children: localisation acuity measured with minimum audible angle. Ear and Hearing, 2006 27(1): p. 43–59. 10.1097/01.aud.0000194515.28023.4b 16446564 PMC2651156

[25] Litovsky R., Johnstone P.M., and Godar S.P., Benefits of bilateral cochlear implants and/or hearing aids in children. Int J Audiol, 2006 45(Suppl): p. S78–91. 16938779 10.1080/14992020600782956 PMC2644458

[26] Peters B.R., et al, Importance of age and postimplantation experience on speech perception measures in children with sequential bilateral cochlear implants. Otology and Neurotology, 2007 28(5): p. 649–657. 10.1097/01.mao.0000281807.89938.60 17712290

[27] Zeitler D.M., et al, Speech perception benefits of sequential bilateral cochlear implantation in children and adults: A retrospective analysis. Otology and Neurotology, 2008 29(3): p. 314–325. 18494140 10.1097/mao.0b013e3181662cb5

[28] Schafer E. and Thibodeau L.M., Speech recognition in noise in chidren with cochlear implants while listening in bilateral, bimodal, and *FM-system arrangements*. American Journal of Audiology, 2006 15: p. 114–26. 10.1044/1059-0889(2006/015) 17182876

[29] Boons T., et al, Predictors of spoken language development following pediatric cochlear implantation. Ear and hearing, 2012 33(5): p. 617–639. 10.1097/AUD.0b013e3182503e47 22555184

[30] Schafer E.C., et al, A meta-analysis to compare speech recognition in noise with bilateral cochlear implants and bimodal stimulation. Int J Audiol, 2011 50(12): p. 871–80. 10.3109/14992027.2011.622300 22103439

[31] Drummond M.F., et al, *Methods for the Economic Evaluation of Health Care Programmes*. Third ed. 2005, United States: Oxford University Press.

[32] Broomfield S.J., et al, Results of a prospective surgical audit of bilateral paediatric cochlear implantation in the UK. Cochlear Implants Int, 2014 15(5): p. 246–53. 10.1179/1754762813Y.0000000041 24621149

[33] Department of Statistics Singapore, Complete life tables 2014–2015 for Singapore Resident Population. 2016, Department of Statistics Singapore: Singapore.

[34] Avan P., Giraudet F., and Buki B., Importance of binaural hearing. Audiol Neurootol, 2015 20 Suppl 1: p. 3–6. 25998698 10.1159/000380741

[35] Cochlear Limited. Cochlear Nucleus Reliability Report. 2016; Available from: http://www.cochlear.com/wps/wcm/connect/sg/home/discover/cochlear-ipmlants/nucleus-6/cochlears-implant-portfolio/reliability-report.

[36] Advanced Bionics. 2015 Cochlear implant reliability report. 2015; Available from: https://www.advancedbionics.com/content/dam/advancedbionics/Documents/Regional/BR/AB_2015_Cochlear_Implant_Reliability_Report.pdf.

[37] Med-El. Reliability you can count on. 2016; Available from: www.medel.com/reliability-reporting/.

[38] Sharma A., Dorman M.F., and Kral A., The influence of a sensitive period on central auditory development in children with unilateral and bilateral cochlear implants. Hearing Research, 2005 203: p. 134–43. 10.1016/j.heares.2004.12.010 15855038

[39] Barton G.R., et al, Hearing-impaired children in the United Kingdom, IV: cost-effectiveness of pediatric cochlear implantation. Ear Hear, 2006 27(5): p. 575–88. 10.1097/01.aud.0000233967.11072.24 16957506

[40] Quentin Summerfield A., et al, Self-reported benefits from successive bilateral cochlear implantation in post-lingually deafened adults: randomised controlled trial. Int J Audiol, 2006 45 Suppl 1: p. S99–107. 16938781 10.1080/14992020600783079

[41] Perez-Martin J., Artaso M.A., and Diez F.J., Cost-effectiveness of pediatric bilateral cochlear implantation in Spain. Laryngoscope, 2017 127(12): p. 2866–2872. 10.1002/lary.26765 28776715

[42] Summerfield A.Q., et al, Estimates of the cost-effectiveness of pediatric bilateral cochlear implantation. Ear Hear, 2010 31(5): p. 611–24. 10.1097/AUD.0b013e3181de40cd 20473177

[43] Bond M., et al, Effectiveness of multi-channel unilateral cochlear implants for profoundly deaf children: a systematic review. Clin Otolaryngol, 2009 34(3): p. 199–211. 10.1111/j.1749-4486.2009.01916.x 19531168

[44] Lammers M.J., et al, Bilateral cochlear implantation in children: a systematic review and best-evidence synthesis. Laryngoscope, 2014 124(7): p. 1694–9. 10.1002/lary.24582 24390811

[45] Advanced Bionics. The Naida bomodal hearing solution. 2018 [cited 2018 19 Feb]; Available from: https://www.advancedbionics.com/com/en/home/products/naida-bimodal-hearing-solution.html.

[46] Oticon. Oticon medical streamer bimodal guide. 2018 [cited 2018 19 Feb]; Available from: https://www.oticonmedical.com/-/media/medical/main/files/bahs/products/om-streamer/ifu/eng/oticon-medical-streamer-bimodal-guide—english—m52707.pdf?la=en.

