# Supplementary material for: Cost-effectiveness analysis of bilateral cochlear implants for children with severe-to-profound sensorineural hearing loss in both ears in Singapore
Source: PLoS One. 2019 Aug 15;14(8):e0220439. doi: 10.1371/journal.pone.0220439 (PMC6695111; doi:10.1371/journal.pone.0220439)
Supplement: S1 File — (DOCX) [file pone.0220439.s001.docx]

| Model | N | Weight | Function | Parameters | | 1 | 2 | 3 | 4 | 5 | 6 | 7 | 8 | 9 | 10 | 11 | 12 | 13 | 14 | 15 | 16 | 17 | 18 | 19 |
| --- | --- | --- | --- | --- | --- | --- | --- | --- | --- | --- | --- | --- | --- | --- | --- | --- | --- | --- | --- | --- | --- | --- | --- | --- |
| HiRes 90K (Vendor A Post Mod) | 25513 | 0.113386 | Weibull | 0.002 | 1.160 | 0.998 | 0.996 | 0.993 | 0.991 | 0.988 | 0.985 | 0.982 | 0.980 | 0.977 | 0.974 | 0.971 | 0.968 | 0.964 | 0.961 | 0.958 | 0.955 | 0.952 | 0.949 | 0.945 |
| HiRes 90K (Vendor A All) | 26605 | 0.118239 | Weibull | 0.002 | 1.191 | 0.998 | 0.996 | 0.993 | 0.990 | 0.987 | 0.984 | 0.981 | 0.977 | 0.974 | 0.971 | 0.967 | 0.964 | 0.960 | 0.956 | 0.953 | 0.949 | 0.945 | 0.942 | 0.938 |
| HiRes 90K (Vendor A Pre Mod) | 1092 | 0.004853 | Weibull | 0.005 | 1.118 | 0.995 | 0.990 | 0.985 | 0.979 | 0.973 | 0.967 | 0.961 | 0.954 | 0.948 | 0.942 | 0.936 | 0.929 | 0.923 | 0.917 | 0.910 | 0.904 | 0.897 | 0.891 | 0.885 |
| HiRes 90K (Vendor B) | 1843 | 0.008191 | Weibull | 0.035 | 1.210 | 0.966 | 0.923 | 0.878 | 0.831 | 0.785 | 0.739 | 0.695 | 0.652 | 0.611 | 0.571 | 0.533 | 0.497 | 0.463 | 0.431 | 0.401 | 0.372 | 0.345 | 0.320 | 0.296 |
| Clarion CII | 2079 | 0.00924 | Weibull | 0.007 | 0.757 | 0.993 | 0.988 | 0.984 | 0.980 | 0.976 | 0.972 | 0.969 | 0.966 | 0.963 | 0.960 | 0.957 | 0.954 | 0.951 | 0.948 | 0.945 | 0.943 | 0.940 | 0.938 | 0.935 |
| Clarion 1.2 | 4149 | 0.018439 | Weibull | 0.013 | 1.116 | 0.988 | 0.973 | 0.958 | 0.943 | 0.927 | 0.911 | 0.896 | 0.880 | 0.864 | 0.849 | 0.833 | 0.818 | 0.803 | 0.788 | 0.773 | 0.758 | 0.743 | 0.729 | 0.715 |
| CI24RE series | 84440 | 0.375271 | Weibull | 0.002 | 0.786 | 0.998 | 0.997 | 0.995 | 0.994 | 0.993 | 0.992 | 0.991 | 0.990 | 0.989 | 0.988 | 0.987 | 0.986 | 0.985 | 0.985 | 0.984 | 0.983 | 0.982 | 0.981 | 0.981 |
| CI500 Series | 14388 | 0.063944 | Weibull | 0.039 | 0.524 | 0.962 | 0.945 | 0.933 | 0.922 | 0.913 | 0.905 | 0.897 | 0.890 | 0.884 | 0.878 | 0.872 | 0.866 | 0.861 | 0.856 | 0.851 | 0.846 | 0.842 | 0.837 | 0.833 |
| CI24R | 34521 | 0.153419 | Weibull | 0.003 | 0.806 | 0.997 | 0.994 | 0.992 | 0.990 | 0.988 | 0.986 | 0.984 | 0.982 | 0.981 | 0.979 | 0.977 | 0.976 | 0.974 | 0.972 | 0.971 | 0.969 | 0.968 | 0.966 | 0.965 |
| CI24M | 11824 | 0.052549 | Weibull | 0.011 | 0.553 | 0.989 | 0.984 | 0.980 | 0.977 | 0.974 | 0.971 | 0.968 | 0.966 | 0.964 | 0.961 | 0.959 | 0.957 | 0.956 | 0.954 | 0.952 | 0.950 | 0.949 | 0.947 | 0.945 |
| CI22M | 8224 | 0.036549 | Weibull | 0.010 | 0.713 | 0.990 | 0.983 | 0.977 | 0.972 | 0.968 | 0.963 | 0.959 | 0.955 | 0.951 | 0.947 | 0.944 | 0.940 | 0.937 | 0.934 | 0.930 | 0.927 | 0.924 | 0.921 | 0.918 |
| Digisonic SP | 8688 | 0.038611 | Weibull | 0.006 | 0.754 | 0.994 | 0.990 | 0.986 | 0.983 | 0.980 | 0.977 | 0.974 | 0.971 | 0.968 | 0.966 | 0.963 | 0.961 | 0.958 | 0.956 | 0.954 | 0.952 | 0.949 | 0.947 | 0.945 |
| Digisonic SP EVO | 1645 | 0.007311 | Gompertz | 0.00001 | 3.161 | 1.000 | 1.000 | 1.000 | 0.999 | 0.998 | 0.997 | 0.995 | 0.993 | 0.990 | 0.986 | 0.981 | 0.975 | 0.967 | 0.959 | 0.949 | 0.938 | 0.925 | 0.911 | 0.896 |
| Pooled | 225011 | 1 | Not applicable | Not applicable | Not applicable | 0.994 | 0.990 | 0.987 | 0.983 | 0.980 | 0.977 | 0.974 | 0.971 | 0.969 | 0.966 | 0.963 | 0.960 | 0.958 | 0.955 | 0.952 | 0.950 | 0.947 | 0.945 | 0.942 |

**Pooled empirical survival data of internal cochlear implant device from the manufacturers’ cochlear implant reliability reports**
